# Differential Infectivity of Human Neural Cell Lines by a Dengue Virus Serotype-3 Genotype-III with a Distinct Nonstructural Protein 2A (NS2A) Amino Acid Substitution Isolated from the Cerebrospinal Fluid of a Dengue Encephalitis Patient

**DOI:** 10.1155/2023/2635383

**Published:** 2023-01-17

**Authors:** Minh Huong Phu Ly, Co Thach Nguyen, Thanh Vu Nguyen, Thanh Thi Ngan Nguyen, Takeshi Nabeshima, Ferdinard Adungo, Yuki Takamatsu, Nguyen Tien Huy, Thi Quynh Mai Le, Kouichi Morita, Futoshi Hasebe, Meng Ling Moi

**Affiliations:** ^1^Institute of Tropical Medicine, Nagasaki University, Nagasaki, Japan; ^2^National Institute of Hygiene and Epidemiology, Hanoi, Vietnam; ^3^Kenya Medical Research Institute (KEMRI), Nairobi, Kenya; ^4^Graduate School of Medicine, The University of Tokyo, Tokyo, Japan

## Abstract

Dengue encephalitis is considered as a severe but unusual clinical presentation of dengue infection. Limited molecular information is available on the neurotropism of dengue virus (DENV), highlighting the need for further research. During a dengue outbreak in Vietnam in 2013, two DENV-3 strains were isolated, in which one was isolated from cerebrospinal fluid (CSF) samples from a dengue encephalitis patient and another strain was isolated from a patient with classical dengue fever in Hai Phong, Vietnam. DENV serotype-3 (DENV-3) isolated from these samples belonged to genotype III, marking the first report of this genotype in the country at that time. Genetic variation between both strains was elucidated by using a full genome sequencing by next-generation sequencing (NGS). The infectivity of the isolated DENV-3 strains was further characterized using human and mouse neuronal cell lines. Phylogenetic analysis of the isolates demonstrated high homogeneity between the CSF-derived and serum-derived DENV-3, in which the full genome sequences of the CSF-derived DENV-3 presented a *Thr-1339-Ile* mutation in the nonstructural 2A (NS2A) protein. The CSF-derived DENV-3 isolate grew preferentially in human neuronal cells, with a significant proportion of cells that were positive for nonstructural 1 (NS1), nonstructural 4B (NS4B), and nonstructural 5 (NS5) antigens. These results suggest that NS2A may be a crucial region in the neuropathogenesis of DENV-3 and its growth in human neuronal cells. Taken together, our results demonstrate that a CSF-derived DENV-3 has unique infectivity characteristics for human neuronal cells, which might play a crucial role in the neuropathogenesis of DENV infection.

## 1. Introduction

Dengue is an acute mosquito-borne viral disease, and its global incidence has grown dramatically in recent decades. The disease places a notable socioeconomic burden on many tropical and subtropical regions of the world [1]. It is estimated that more than 390 million dengue virus (DENV) infections occur annually, and approximately 96 million cases are symptomatic, accounting for 2.5% of the deaths among hospitalized cases [1]. DENV is a single positive-stranded RNA virus that belongs to the family *Flaviviridae* and genus *Flavivirus*. There are four DENV serotypes (DENV-1, DENV-2, DENV-3, and DENV-4), all of which have been individually found to be responsible for dengue epidemics and associated with severe dengue cases [2]. The virus is transmitted by female mosquitoes, mainly *Aedes aegypti*, and to a lesser extent by *Aedes albopictus.* The resurgence of the mosquito vector *Aedes aegypti,* overcrowding, and increasing travel have been related to the expansion of dengue infection throughout the world [2, 3].

The first neurological involvement in dengue infection was described by Sanguansermsri et al. in 1976 [3–5]. Despite not being categorized as a neurotropic virus, DENV can cause neurological manifestations that can be classified into three categories based on the pathogenesis: (1) metabolic disturbance, for example, encephalopathy; (2) viral invasion, including encephalitis, meningitis, and myelitis; and (3) autoimmune reactions, including disseminated encephalomyelitis, neuromyelitis optica, optic neuritis, myelitis, encephalopathy, and Guillain–Barré syndrome [6, 7]. These clinical features have long been observed among dengue infections, although they received little attention initially. Neurological symptoms manifest in individuals whose age varies from 3 months to 60 years old and have an associated incidence and mortality of 0.5–21% and 5–8%, respectively [6, 8–10]. Neuropathogenesis has been mainly associated with DENV-2 and DENV-3 serotypes [11, 12]. Additionally, DENV-4 has also been detected in human neural cells by immunohistochemistry and in the cerebrospinal fluid (CSF) of a patient with encephalitis [13–15]. In some cases, neurological involvement may be the first manifestation of DENV infection [16, 17]. In addition, 20–30% of patients with dengue encephalitis manifest sequelae, including spastic paraparesis associated with myelitis, mental confusion, and personality changes [8, 18].

Although the incidence of dengue cases presenting with neurological impairments continues to increase during dengue epidemics worldwide, the neuropathogenesis of DENV infection is still poorly understood. It is known that virus and host factors play an important role in the neurological disorders associated with dengue, as more evidence strongly supports the notion that the dengue virus is directly neurovirulent, although it is not classically categorized as a neurotropic pathogen. Here, we determined the *in vitro* infectivity of the two DENV-3 strains using human neural cell lines. One strain was isolated from the cerebrospinal fluid (CSF) of a patient who was diagnosed with dengue encephalitis, and another strain was isolated from serum of a patient with classical dengue fever in Hai Phong, Vietnam, in 2013.

## 2. Materials and Methods

### 2.1. Patient Samples

Cerebrospinal fluid (CSF) specimens were obtained from a single dengue encephalitis patient in Hai Phong, Vietnam, in 2013. Two serum samples from two different patients with classical dengue fever were also obtained during the dengue endemic season in Hai Phong, Vietnam, in 2013 (Supplementary Figures 1 and 2). The virus strains isolated from clinical samples were used in the subsequent analysis.

### 2.2. Virus and Cell Line

To isolate the virus, 10 *μ*l of CSF and serum specimens were inoculated onto C6/36 mosquito cells and Vero cells (African green monkey epithelial kidney ATCC, CCL81). Vero cells were maintained at 37°C and C6/36 at 28°C in Eagle's minimum essential medium (MEM), containing 10% fetal bovine serum (FBS) and 0.2 mM nonessential amino acids. Cells were observed daily for the cell cytopathic effect (CPE) for a total of one week after infection. Viral RNA was extracted from infected culture fluids on day 7, by using a QIAamp Viral RNA Mini Kit (QIAGEN), and the presence of DENV-3 RNA was confirmed by RT-PCR [19]. Undiluted virus stock (i.e., infected culture fluids) was stored at −80°C until use. The virus isolates were used within five passages and maintained in C6/36 cells throughout the study.

In this study, human neuroblastoma SKNSH cells (ACTT®-HTB-11™), human glioblastoma T98G cells (ACTT®-CRL-1690), and murine neuroblastoma N2A cells (ACTT®-CCL-131) were used to determine the infectivity of the virus isolates from CSF specimens and serum samples. The cells were maintained and cultured according to the manufacturer's instructions.

### 2.3. Sequence and Phylogenetic Analyses

Due to the limited amount of CSF and serum specimens, samples were initially inoculated onto C6/36 mosquito and Vero cells. RNA was extracted from infected culture fluids using the QIAamp Viral RNA Mini Kit (QIAGEN), and the presence of DENV-3 RNA was confirmed by RT-PCR. The RNA sequence was analyzed using an Ion Proton (Thermo Fisher) and a conventional capillary sequencer (3100-Avant Genetic Analyzer, Life Technologies). Nucleotide sequences were aligned using the MAFFT software version 7.215 [20]. The substitution models were selected using jModelTest-2.1.7 [21], and GTR + I + G was used as the model. The phylogenetic tree was constructed using FigTree software, version 1.4.0.

### 2.4. Focus Forming Assay

CSF-derived DENV-3 and serum-derived DENV-3 strains were separately inoculated onto mosquito cells C6/36, SKNSH, T98G, and N2A cell lines at MOI of 0.1, 0.01, and 0.001, respectively. The supernatant and infected cells were separately harvested at 0, 24, 48, 72, 96, and 120 h postinfection. Next, the infected cells were lysed with the RNeasy Plus Mini Kit (QIAGEN) for RNA extraction. Both supernatant and virus RNA from infected cells were stored at −80°C for further examination. A virus titration was performed using the cell culture supernatant by a focus forming assay on Vero cells. Briefly, the culture supernatant was serially diluted (10-fold) in MEM containing 2% fetal calf serum, and 100 *μ*l of each dilution was inoculated onto a Vero cell monolayer in a 96-well plate and incubated at 37°C for one hour. The infected cells were then overlaid with MEM containing 2% fetal calf serum and 1.25% methylcellulose. After a four-day incubation at 37°C, the plates were fixed with 4% paraformaldehyde phosphate buffer solution (Wako, Osaka, Japan) for 30 min at RT, rinsed, and then permeabilized with 100 *μ*l of 1% NP-40 solution in PBS (1×) per well for 30 min at RT. The plates were again washed three times with PBS (1×) and then blocked with undiluted blockage (UK-B 80, Yukijirushi, Sapporo, Japan) at 4°C overnight. Focus immunostaining was conducted after the blocking step, and 100 *μ*l of anti-Flavivirus mAb 12D11/7E8 [22] cell supernatant was applied to each well and incubated for one hour at 37°C. The plates were washed three times with PBS (1×). A total of 100 *μ*l of HRPO-conjugated goat anti-mouse IgG (American Qualex) diluted to 1 : 500 in blocking buffer was added to each well and incubated for one hour at 37°C. The plates were washed three times with PBS (1×) and 100 *μ*l/well of 0.5 mg/ml solution of substrate 3,3′-diaminobenzidine tetrahydrochloride (DAB, Wako) in PBS (1×) with 0.03% H_2_O_2_ was added to each well and incubated for 30 min at RT. The plates were finally washed with distilled water, and the number of foci per well was counted under a microscope.

### 2.5. Dengue Virus Quantification by Real-Time PCR

To determine the intracellular copy number of DENV-3 RNA in infected cells, viral RNA was extracted from infected cells using the RNeasy® Plus Mini Kit (QIAGEN) according to the manufacturer's instructions, and the expression level of the mRNA encoding the DENV-3 envelope protein was determined using real-time PCR (QuantStudio 7 Flex Real-Time PCR System, Life Technologies). Briefly, 5 *μ*L of purified RNA was amplified in a 20 *μ*L reaction mix containing 250 nM TaqMan® MGB Probe Real-Time PCR (5′-FAM-AGATTTTGTGGAAGGYCT-3′), 5 *μ*L of 4× TaqMan® Fast Virus 1-Step Master Mix (Life Technology, Foster, CA), 700 nM forward primer (5′-CYTGGWTGTCDRCYGARGGAG), 700 nM reverse primer (5′-TGCACCACTTTTCCCTCTAT-3′), and 6.7 *μ*L of distilled water (Supplementary Tables 1 and 2). Reactions were incubated at 50°C for 5 min, followed by incubation at 95°C for 20 s and 40 cycles of 95°C for 3 s and 60°C for 30 s.

To generate a standard curve, a region of 545 base pairs from the envelope sequence of DENV-3 was first amplified by forward primer 5′-CYTGGWTGTCDRCYGARGGAG-3′ and reverse primer 5′-TGCACCACTTTTCCCTCTAT-3′, then cloned into TOPO TA cloning vector (Invitrogen). The plasmid of a positive colony was extracted, linearized, and then transcribed using the T7 mMessage mMachine kit (Ambion). The *in vitro* transcribed RNA was diluted in a series of 10-fold dilutions equivalent to 10^7^–10^3^ copies/reaction to generate the standard curve. This 10-fold standard curve was run in parallel with the test samples in the 96-microplate. The copy number of the DENV-3 RNA from infected cells was measured based on the standard curve.

### 2.6. Immunofluorescence Microscopy Assay (IFA)

An immunofluorescence microscopy assay was used to determine the infectivity of the CSF-derived DENV-3 and serum-derived DENV-3 strains in human neuroblastoma (SKNSH), glioblastoma (T98G), and mouse neuroblastoma cell lines (N2A). For this purpose, the different cell lines were infected with either CSF-derived DENV-3 or serum-derived DENV-3 at a multiplicity of infection (MOI) of 0.1, 0.01, and 0.001. The infected cells were harvested on day 2 postinfection (P.I) and centrifuged at 100 rpm for 5 min. The cell pellet was washed three times with PBS (1×) and applied onto a Teflon-coated eight-multiwell glass slide (MP Biochemicals, CA, USA). After complete drying, the cells were fixed for 10 min in a fixative solution consisting of methanol and acetone at a ratio of 1 : 1 at room temperature (RT). The slides were then washed three times in PBS (1×) with gentle shaking and then air-dried for 10 min at RT. For each test well, 15 *μ*l of rabbit polyclonal antibody against the DENV envelope protein and nonstructural proteins 1–5, except NS2A (GeneTex, USA), was diluted 1 : 100 in PBS (1×). Samples were incubated with each of the diluted antibody mixtures for one hour in a moist chamber at 37°C. Anti-Flavivirus monoclonal antibody 12D11/7E8 [22] was used as a positive control. The slides were washed three times in PBS (1×) and air-dried for 10 min at RT. Next, 15 *μ*l of goat anti-rabbit IgG H&L (ab150077–ALexa Fluor® 488, Japan) and 15 *μ*l of goat anti-mouse IgG H&L (ab150113, ALexa Fluor® 488, Japan) at a dilution of 1 : 50 were applied to each test well and to the positive control well, respectively, and incubated for one hour at 37°C in the dark. The slides were washed three times in PBS (1×) and air-dried for 10 min and finally examined by fluorescence microscopy.

### 2.7. Data Analysis

In this study, all data from the focus forming assay and real-time PCR were transformed to a base-10 logarithm for analysis. In descriptive analyses, numbers and percentages were used for categorical variables (percentage of infected cells in different protein expressions from an envelope, nonstructural 1–5). The mean and standard deviation (SD) were used for continuous variables (viral load at 0, 24, 48, 96, and 120 h). For comparison in specific groups, the chi-squared test, Fisher's exact test, Wilcoxon rank-sum (Mann–Whitney) test, and *t*-test were used appropriately. Statistical tests were performed using Stata 14.1 (StataCorp LP, College Station, Texas 77845, USA) and GraphPad Prism version 7.0a (GraphPad software, La Jolla, California, USA) with a 5% level of significance and two-tailed *p* values.

## 3. Results

### 3.1. Phylogenetic Analysis and Comparison of Amino Acid Sequences

The complete envelope nucleotide sequences of the CSF-derived DENV-3 isolate (accession no. KP893717) and two serum-derived DENV-3 isolates from patients with classical dengue fever in Hai Phong, Vietnam, in 2013 (accession nos. KP893718 and KP893719) were determined and deposited in GenBank [19]. The envelope sequences available in GenBank corresponding to strains that belong to the major branches of DENV-3 phylogenies were included in the analysis. Envelope nucleotide sequences were initially aligned using MAFFT version 7.215 [20], and the substitution models were selected by jModelTest-2.1.7 [21]. GTR + I + G was used as the model to construct the phylogenetic trees using the maximum likelihood method using PHYML 3.0.1 [23]. Phylogenetic analysis showed a close relationship between CSF-derived DENV-3 and the two serum-derived DENV-3 strains. All of the isolates belonged to DENV-3 genotype-III (Figure S1). DENV-3 genotype II was found to circulate in Vietnam in 2003, 2006, 2007, and 2009. DENV-3 genotype-III was first reported in the Indian subcontinent since the 1960s [24] and then in Sri Lanka in the 1980s and the 1990s. Since the 2000s, the number of dengue cases caused by this genotype has substantially increased in many countries, including Cambodia, Bhutan, Thailand, Laos, Pakistan, China, Senegal, and Singapore [24–28]. According to the national data report in 2013, two other dengue cases isolated in the Ha Tinh province were determined to be caused by DENV-3 genotype-III (Figure S1). Therefore, it is possible that the emergence of DENV-3 genotype-III in Vietnam occurred before 2013 and spread throughout the region, including Hai Phong, but also the neighboring areas in the north of Vietnam by 2013.

Next, we determined the full-length genome sequence of the DENV-3 strains isolated from serum and CSF samples and mapped the amino acid differences between the strains [19]. The three strains did not have any significant differences in the nucleotide and amino acid substitutions in the envelope sequence [19]. However, full genome analyses demonstrated a *Thr-1339-Ile* mutation (threonine was substituted by isoleucine) in the nonstructural region 2A of the CSF-derived DENV-3 (Figure S2) and an *Ala-3018-Thr* mutation (alanine was substituted by threonine) in the nonstructural region 5 of the serum-derived strain (HP-5528) (Figure S2). In this study, the infectivity and phenotypes of the two DENV-3 strains, a serum-derived DENV-3 strain (HP-5528) and a CSF-derived DENV-3 strain (CSF-11098), were determined *in vitro* using mosquito cell C6/36 and human and mouse neuronal cell lines.

### 3.2. DENV Infectivity in Neuronal Cell Lines as Determined by Focus Forming Assay (FFA)

**Figure 1:**
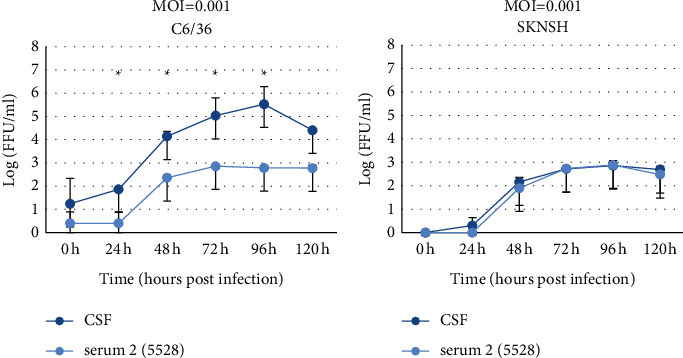
DENV-3 growth curve of two different isolates, cerebrospinal fluid (CSF)- and serum-derived DENV-3 genotype-III, using a mosquito cell line (C6/36) and a human neural cell line (SKNSH). After infection at a multiplicity of infection (MOI) = 0.001, cell culture fluids were collected every 24 hours from 0 to 120 h and titrated by using a focus forming assay on Vero cells. *P* value was determined using the *t* test. *P* value ≤0.05 was considered significant.

To determine the infectivity of the isolates, a focus forming assay on the Vero cell was performed to determine the virus titer in each cell line. DENV-3 foci were observed in cell culture supernatant harvested from a mosquito cell line (C6/36) at three different MOIs (0.1, 0.01, and 0.001) and from human neuroblastoma cells (SKNSH) at MOI = 0.001. However, we did not observe any focus when infecting culture supernatant harvested from human glioblastoma (T98G) and mouse neuroblastoma (N2A) cells were inoculated on Vero cells at MOI of 0.01 and 0.001. As shown in Figure 1(b). virus titers at 24 h and 48 h P.I were higher in infected culture fluids (ICF) from CSF-derived DENV-3 as compared to ICF from serum-derived DENV-3, although the results were not statistically significant. Interestingly, in a mosquito cell line, the virus titers in the culture supernatant from CSF-derived DENV-3 at 24 h, 48 h, 72 h, and 96 h P.I were higher at 1.86 ± 0.06 (mean ± SD), 4.14 ± 0.21, 5.03 ± 0.77, and 5.53 ± 0.76, respectively (Figure 1(a)), while the virus titers in cell culture supernatant from serum-derived DENV-3 (5528) at the same time points were 0.4 ± 0.49, 2.36 ± 0.12, 2.85 ± 0.03, and 2.78 ± 0.09, respectively (Figure 1(a)). The mean virus titer for CSF-derived DENV-3 was significantly higher than that of serum-derived DENV-3 (*p* < 0.05) (Figure 1(a)).

### 3.3. Quantification of DENV-3 Viral RNA Levels in Infected Cells by Real-Time PCR

**Figure 2:**
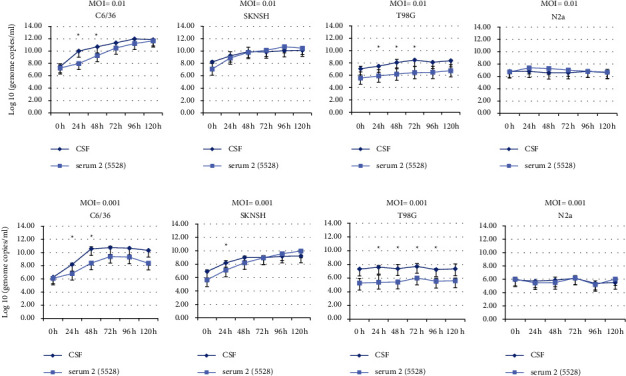
DENV-3 genome levels at 0, 24, 48, 72, 96, and 120 h postinfection (P.I). Virus genome levels were quantified by using real-time qPCR after infection at a multiplicity of infection (MOI) = 0.01 ((a)–(d)) and MOI = 0.001 ((e)–(h)). DENV-3 isolated from the cerebrospinal fluid (CSF) specimen (CSF-11098) and serum specimen (HP-5528) was separately inoculated to C6/36, SKNSH, T98G, and N2A cell lines. Viral genome copies number present in infected cells was determined at different time points and compared between a CSF sample and a serum sample. *P* value was determined by the *t*-test. ^*∗*^*P* value ≤0.05 was considered significant.

**Figure 3:**
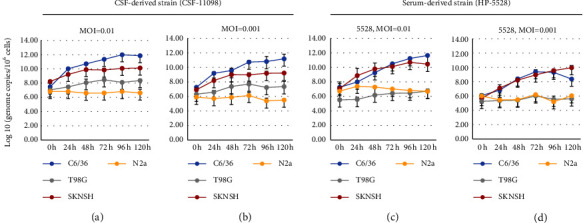
DENV-3 RNA genome levels as determined from infected cells at 0, 24, 48, 72, 96, and 120 h postinfection determined by RT-qPCR at a multiplicity of infection (MOI) of 0.01 and 0.001. DENV-3 isolated from the cerebrospinal fluid (CSF) specimen (CSF-11098) and the serum specimen (HP-5528) was separately inoculated to C6/36, SKNSH, T98G, and N2A cell lines. *P* value was determined by the *t*-test. ^*∗*^*P* value ≤0.05 was considered significant.

CSF-derived DENV-3 (CSF-11098) and serum-derived DENV-3 (HP-5528) strains were separately inoculated onto mosquito cells C6/36, SKNSH, T98G, and N2A cell lines at MOI of 0.1, 0.01, and 0.001, respectively. Infected cells were harvested at different time points P.I. (0, 24, 48, 72, 96, and 120 h), lysed, and RNA was extracted. The intracellular copy number of DENV-3 RNA molecules was measured at the different time points to compare the probability of infection. At the higher MOI (MOI = 0.01), the virus titers measured at 24 and 48 h P.I in the mosquito cell line (C6/36) were 10.01 ± 0.13 (log10 genome copies/ml, mean_CSF_24h_ ± s.d.) and 10.70 ± 0.04 for CSF-derived DENV-3 (CSF-11098), respectively (Figure 2(a)). For serum-derived DENV-3, the virus titers at 24 and 48 h P.I were mean_serum_24h_ = 8.00 ± 0.19 and mean_serum_48h_ = 9.27 ± 0.00, respectively (Figure 2(a)). The virus titers at these two time points were significantly higher in the CSF-derived DENV-3 (CSF-11098) than in the serum-derived DENV-3 (HP-5528) (*p* < 0.05) (Figure 2(a)). The same pattern of virus titer was also observed when these two isolates were inoculated at a lower MOI (MOI = 0.001) onto the C6/36 cell line (Figure 2(e)). In a human glioblastoma cell line (T98G), the CSF-derived DENV-3 isolate demonstrated a consistently increased growth pattern compared to the serum-derived DENV-3 isolate (HP-5528) (Figures 2(c) and 2(g)). The virus titers were approximately 10 times higher at 24, 48, and 72 h P.I. for CSF-derived DENV-3 (7.47 ± 0.04, 8.06 ± 0.51, and 8.46 ± 0.04, respectively) (Figure 2(c)). For serum-derived DENV-3 (HP-5528), the virus titers at 24, 48, and 72 h P.I. were 5.88 ± 0.5, 6.19 ± 0.04, and 6.43 ± 0.09, respectively (*p* < 0.05) (Figure 2(c)). There was no significant difference in virus titer between the CSF-derived DENV-3 isolate (CSF-11098) and the serum-derived DENV-3 isolate (HP-5528) when these viruses were inoculated to human neuroblastoma (SKNSH) cell line at MOI 0.01 (Figure 2(b)). However, at a lower MOI (MOI = 0.001), in the human neuroblastoma cell line (SKNSH), the virus copy number of the CSF-derived DENV-3 isolate was higher than that of the serum-derived DENV-3 isolate (HP-5528) only at 24 h P.I (8.19 ± 0.36 vs. 7.12 ± 0.44, *p* < 0.05) (Figure 2(f)). However, neither CSF-derived nor serum-derived DENV-3 demonstrated growth in mouse neural cells (N2a) at either MOI (Figures 2(d) and 2(h)). DENV-3 infectivity among the four cell lines (C6/36, SKNSH, T98G, and N2A) was further assessed using the CSF-derived DENV-3 isolate and the serum-derived DENV-3 isolate (HP-5528). Overall, the mosquito cells (C6/36) and human neuroblastoma cells (SKNSH) presented higher virus titers than the two other cell lines (T98G and N2A), regardless of the initial MOI (Figure 3). The virus titer was approximately one log higher than that of the initial inoculation for CSF-derived DENV-3 in T98G cells (Figures 3(a) and 3(b)). The serum-derived DENV-3 strain, however, did not demonstrate a clear increase in virus titers during an incubation period up to 120 h in both T98G and N2A cells (Figures 3(c) and 3(d)).

### 3.4. Levels of Viral Structural and Nonstructural Proteins in DENV-3 Infected Human Neural Cell Lines

**Figure 4:**
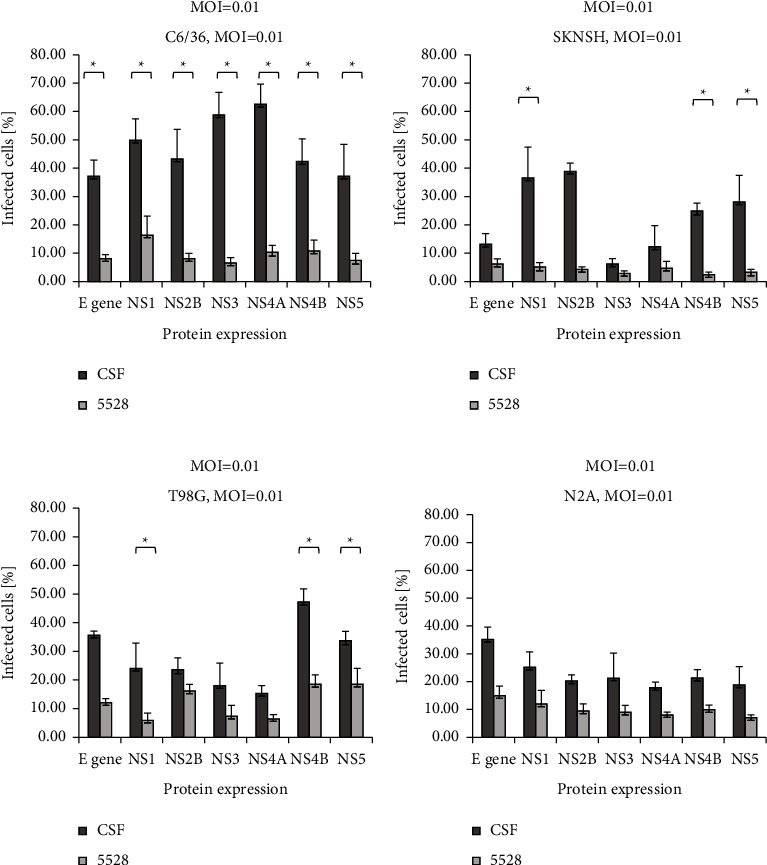
Infectivity of DENV-3 was measured by the expression of viral antigen-positive cells in mosquito and neural cell lines. DENV-3 antigen-positive cells are shown as determined by using immunofluorescence (IFA) staining at 48 hours postinfection at a multiplicity of infection (MOI) of 0.01. DENV-3 isolated from the cerebrospinal fluid (CSF) specimen (CSF-11098) and the serum specimen (HP-5528) were separately inoculated to C6/36, SKNSH, T98G, and N2A cell lines, and viral antigen (E NS1, NS2B, NS3, NS4A, NS4B, and NS5) were detected, respectively, by using IFA. Infected cells (%) were determined by average values (infected/total infected and noninfected cells) × 100% in three random fields at a magnification 200x. *P* value was determined by the *t*-test. ^*∗*^*P* value ≤0.05 was considered significant.

**Figure 5:**
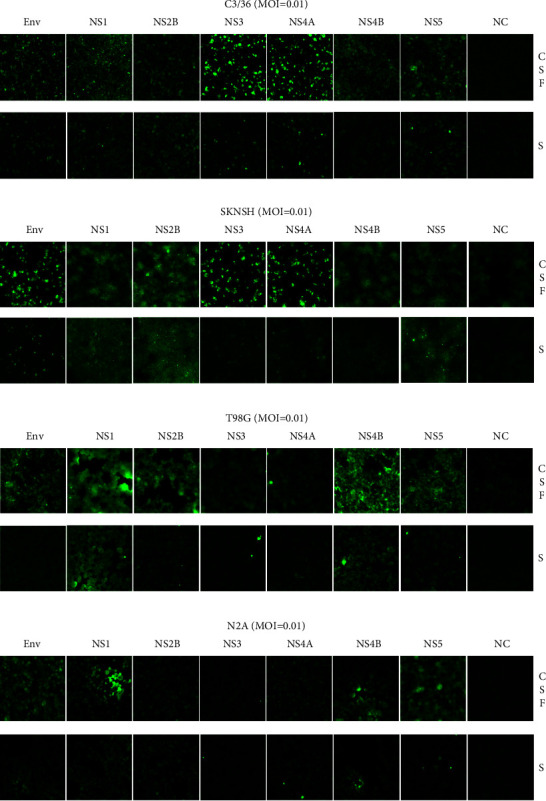
Infectivity of DENV-3 in mosquito and neural cell lines. DENV-3 antigen against structural (E) and nonstructural antigens (NS1, NS2B, NS3, NS4A, NS4B, and NS5) is shown as determined by using immunofluorescence (IFA) staining at 48 hours postinfection at a multiplicity of infection (MOI) of 0.01. DENV-3 isolated from the cerebrospinal fluid (CSF) specimen (CSF-11098, indicated as CSF) and the serum specimen (HP-5528, indicated as S) was separately inoculated to the C6/36, SKNSH, T98G, and N2A cell lines. Each figure represents a random field at a 200x magnification.

The infectivity of the CSF-derived DENV-3 isolate (CSF-11098) and serum-derived DENV-3 isolate (HP-5528) in different neural cell lines was determined by measuring the proportions of cells presenting structural (envelope) and nonstructural proteins (NS1, NS2B, NS3, NS4A, NS4B, and NS5) (Figures 4 and 5). All infected cells were harvested 48 h P.I. At MOI = 0.01, the percentage of C6/36 cells infected with CSF-derived DENV-3 was significantly higher than that of cells infected with serum-derived DENV-3 (Figure 4(a)) (*p* < 0.05). In human neural cells (SKNSH and T98G), the proportion of infected cells detected by antibodies against NS1, NS4B, and NS5 was significantly higher in the group infected with the CSF-derived strain than in that infected with the serum-derived strain (Figures 4(b) and 4(c)) (*p* < 0.05). However, there were no significant differences in the infected cells between the CSF- and serum-derived isolates in the mouse neural cell line (Figure 4(d)) across the panel of antibodies used. The results indicate that while virus growth is supported in human neural cell lines, surface antigens may be expressed differentially between CSF- and serum-derived isolates.

## 4. Discussion

Dengue virus infection is a major public health issue affecting almost every country in the tropical and subtropical regions of the world. In the past two decades, the neurological manifestation of dengue has been increasingly recognized due to its severity and sequelae. In most cases, the neurological involvement of dengue manifests through conditions such as mononeuropathies, polyneuropathies, and Guillain–Barré syndrome. Since 2009, the World Health Organization has recategorized CNS involvement as severe dengue [18]. To date, CNS involvement in DENV has been reported in 25 countries spanning almost all continents [29].

The pathogenic mechanism by which the dengue virus causes neurological manifestations, especially dengue encephalitis, is still poorly understood. In DENV neuropathogenesis, it has been hypothesized that either DENV passively crosses the blood-brain barrier (BBB) and actively invades the CNS system or that the symptoms are the consequence of an opportunistic infection [13]. However, mounting evidence such as the detection of dengue viral RNA in CSF and DENV antigen in brain tissue strongly supports that the virus itself is neurovirulent despite not being classically categorized as a neurotropic pathogen [13, 30, 31]. Moreover, the simultaneous negative findings of viral RNA in CSF and serum samples suggested that the virus might actively enter the CNS rather than passively crossing the BBB [3, 32, 33]. The results of our study of the CSF-derived strain have suggested that mutations within nonstructural proteins could be associated with DENV neuropathogenesis.

In this study, the threonine residue was substituted by the isoleucine residue at position 1339 of the nonstructural 2A glycoprotein in the CSF-derived DENV-3 isolate (CSF-11098). In contrast, there was a substitution of the alanine residue by a threonine at position 3018 in the nonstructural NS5 glycoprotein in the serum-derived DENV-3 isolate (HP-5528). Mutations of three amino acids in the envelope (E) and nonstructural 3 (NS3) gene in DENV-1 produced a neurovirulent phenotype that resulted in extensive brain damage with encephalitis and lepto-meningitis in a mouse model [34]. In 1996, a substitution of alanine by a valine residue at position 173 of the envelope gene was reported in encephalopathy cases associated with a DENV-2 serotype [29]. In addition, Phe-401-Leu and Asp-390-His mutations in the E protein have also been linked as genetic determinants of DENV-2 neurovirulence [35]. Additionally, another study demonstrated that the cleavage of the N153 glycosylation site due to Thr-155-Ile substitution was responsible for the neurovirulence of DENV-4 in mice [36]. Taken together, these studies suggest that the structural protein (envelope) plays a crucial role in the pathogenesis of neuroinvasiveness and neurotropism of the dengue virus. While the CSF-isolate of this study presented none of these reported amino acid substitutions, our results suggest that distinct regions of the NS2A (from CSF-derived-DENV-3) and NS5 proteins (from serum-derived DENV-3) may be associated with the neurovirulence of DENV-3.

In this study, the mutation in the NS5 region in the serum-derived DENV-3 isolate (Supplementary Figure 2) has been hypothesized to be associated with the abrogation of viral replication *in vitro*. A previous study demonstrated that a chimeric virus strain with a mutation in the NS5 gene exhibited comparatively lower growth rates [37]. While differences in the clinical presentation were absent when a mouse model (A129) was inoculated with CSF-derived DENV-3 and serum-derived DENV-3 isolates, further studies are needed to determine the pathogenesis of DENV-related neurological involvement in human patients.

Next, *in vitro* assays were performed to evaluate the infectivity of CSF-derived DENV-3 (CSF-11098) and serum-derived DENV-3 isolates (HP-5528) in human neural cells (SKNSH and T98G) and mouse neuroblastoma cells (N2A). Both isolates propagated well in the human neuroblastoma cell line (SKNSH) compared to the other neural cell lines (T98G and N2A), regardless of the multiplicity of infection (Figure 3). Additionally, the human glioblastoma cell line T98G was a highly permissible cell line for the propagation of the CSF-derived DENV-3 isolate as compared to the serum-derived DENV-3 isolate (Figures 2 and 3).

The neurovascular unit of the BBB consists of astrocytes, pericytes, neurons, and endothelial cells. All of these components function to selectively control the passage of molecules from capillaries into the brain parenchyma and vice versa [38]. The infection of endothelial cells by several types of viruses such as HIV, rabies virus, herpes simplex virus, West Nile virus (WNV), and Japanese encephalitis virus (JEV) is known to cause dramatic changes in BBB function, mainly in permeability and selectively. These changes facilitate plasma leakage and the entry of viruses or infected cells into the brain parenchyma, which promotes the spread of the virus. The brain cell type that is infected by DENV remains controversial. Many studies have demonstrated that DENV is more likely to infect microglial cells, which are macrophage-like resident immune cells in the brain [39]. Another study found that mouse brain endothelial cells (MBECs) were highly susceptible to DENV-4 and neuro-adapted DENV variant infections [38]. In this study, T98G cells were used for the *in vitro* experiment, and these cells exhibited the characteristics of adherent fibroblasts originating from human glioblastoma multiforme. The cellular origin of glioblastoma is unknown, but it has been suggested to be derived from glial cells (astrocytes, oligodendrocytes, microglia, and ependymal) and other neural stem cells. In addition, T98G also expresses the surface markers CD19, CD44, CD90, CD105, and CD133, which are typical of mesenchymal cells [40]. Furthermore, this cell type demonstrated high gene expression of the main angiogenesis inductors vascular endothelial growth factor (VEGF) and fibroblast growth factor 2b (FGF2-b), as well as the matrix protein thrombospondin-1, which is involved in the activation of transforming growth factor *β*1 (TGF*β*1) [40]. The association between these chemokines and the pathogenesis of neurological manifestations in DENV infection requires further study. However, in JEV encephalitis, astrocyte infection results in the release of VEGF, which alters endothelial cell junctions by causing proteasomal degradation of Zonula occludens 1 (ZO-1) [41]. Interestingly, the CSF-derived DENV-3 isolate demonstrated high growth in human neural cell lines (especially in T98G cells), but limited growth was observed in a mouse neuroblastoma cell line, suggesting that the isolate preferentially infected human neural cells. Our data indicate that (1) the CSF-derived DENV-3 strain has unique virulence features potentially associated with the NS2A protein, which might play a crucial role in the neuropathogenesis of DENV infection. The association between the amino acid substitution in the NS2A protein and neuropathogenesis in CSF-derived DENV-3 requires further investigation for elucidation. (2) The mutation in the NS5 protein might restrict the viral replication resulting in a low virus titer of serum-derived DENV-3 in human neural cells as compared to CSF-derived DENV-3. (3) T98G cell line represents a permissible cell line to illuminate the pathogenesis of the DEN-3 serotype and neuro-adapted DENV-3 variants in dengue-related neurological cases.

## 5. Conclusion

We isolated a DENV-3 genotype-III strain from a CSF sample of a DENV encephalitis patient during a DENV epidemic in Vietnam in 2013. The CSF-derived DENV-3 isolate presented enhanced growth in human neuronal cells, with a significant number of cells that were positive for NS1, NS4B, and NS5 antigens. The full-length genome sequence demonstrated that a distinct amino acid substitution in the NS2A protein was unique to the CSF-derived DENV-3 strain. This result suggests that NS2A may be a crucial region in the neuropathogenesis of DENV-3. Taken together, our results demonstrate that a CSF-derived DENV-3 had unique infectivity characteristics for human neuronal cells, which might play a crucial role in the neuropathogenesis of DENV infection. The association between the amino acid substitution in the NS2A protein and neuropathogenesis in CSF-derived DENV-3 requires further investigation.

## Data Availability

The datasets used and analyzed during the current study are available from the corresponding author upon request.

## Abbreviations

BBB: Blood-brain barrier
CNS: Central nervous system
CPE: Cytopathic effect
CSF: Cerebrospinal fluid
DENV: Dengue virus
FCS: Fetal calf serum
FGF2(b): Fibroblast growth factor 2b
IFA: Immunofluorescence microscopy assay
MBECs: Mouse brain endothelial cells
MOI: Multiplicity of infection
NS2A: Nonstructural protein 2A
PBS: Phosphate buffered saline
RT-qPCR: Quantitative real-time polymerase chain reaction
RT: Room temperature
SD: Standard deviation
TGF*β*1: Transforming growth factor *β*1
VEGF: Vascular endothelial growth factor.

## Supplementary Materials

Supplementary Materials Supplementary Figure 1. ML phylogenetic tree based on the envelope gene sequence of DENV-3 isolates. The envelope sequence of DENV-3 (CSF-11098) isolated from the CSF of our case patient in Hai Phong, Vietnam, in 2013 was compared with those of DENV-3 (5228 NGS and 5468 NGS) isolated from serum samples of other patients in the same place and in the same year. The strain names of these three DENV-3 isolates were enclosed in red square. This fragment of CSF-derived DENV-3 was also compared with others homologous sequences of DENV-3 in the GenBank database from different geographical regions. Bootstrap values over 800> of 1000 repeats are shown at the nodes. Labels of strains conform to the following format: (GenBank accession nos)_(Strain name)_(Country-region)_(Year of isolation). Supplementary Figure 2. *Thr-1339-Ile* mutations in nonstructural regions 2A (NS2A) sequences in CSF-derived DENV-3 strain (CSF-11098) and *Ala-3018-Thr* mutation in nonstructural regions 5 (NS5) sequences in serum-derived DENV-3 strain (HP-5528). Supplementary Table 1. Characteristics of the primers and probes used in one-step real-time PCR. Supplementary Table 2. Characteristics of the primers used to generate standard curves for one-step real-time PCR.
