# Supplementary material for: Differential Infectivity of Human Neural Cell Lines by a Dengue Virus Serotype-3 Genotype-III with a Distinct Nonstructural Protein 2A (NS2A) Amino Acid Substitution Isolated from the Cerebrospinal Fluid of a Dengue Encephalitis Patient
Source: Can J Infect Dis Med Microbiol. 2023 Jan 17;2023:2635383. doi: 10.1155/2023/2635383 (PMC9873433; doi:10.1155/2023/2635383)
Supplement: Supplementary Materials — Supplementary Figure 1. ML phylogenetic tree based on the envelope gene sequence of DENV-3 isolates. The envelope sequence of DENV-3 (CSF-11098) isolated from the CSF of our case patient in Hai Phong, Vietnam, in 2013 was compared with those of DENV-3 (5228 NGS and 5468 NGS) isolated from serum samples of other patients in the same place and in the same year. The strain names of these three DENV-3 isolates were enclosed in red square. This fragment of CSF-derived DENV-3 was also compared with others homologous sequences of DENV-3 in the GenBank database from different geographical regions. Bootstrap values over 800> of 1000 repeats are shown at the nodes. Labels of strains conform to the following format: (GenBank accession nos)_(Strain name)_(Country-region)_(Year of isolation). Supplementary Figure 2. Thr-1339-Ile mutations in nonstructural regions 2A (NS2A) sequences in CSF-derived DENV-3 strain (CSF-11098) and Ala-3018-Thr mutation in nonstructural regions 5 (NS5) sequences in serum-derived DENV-3 strain (HP-5528). Supplementary Table 1. Characteristics of the primers and probes used in one-step real-time PCR. Supplementary Table 2. Characteristics of the primers used to generate standard curves for one-step real-time PCR. [file 2635383.f1.docx]

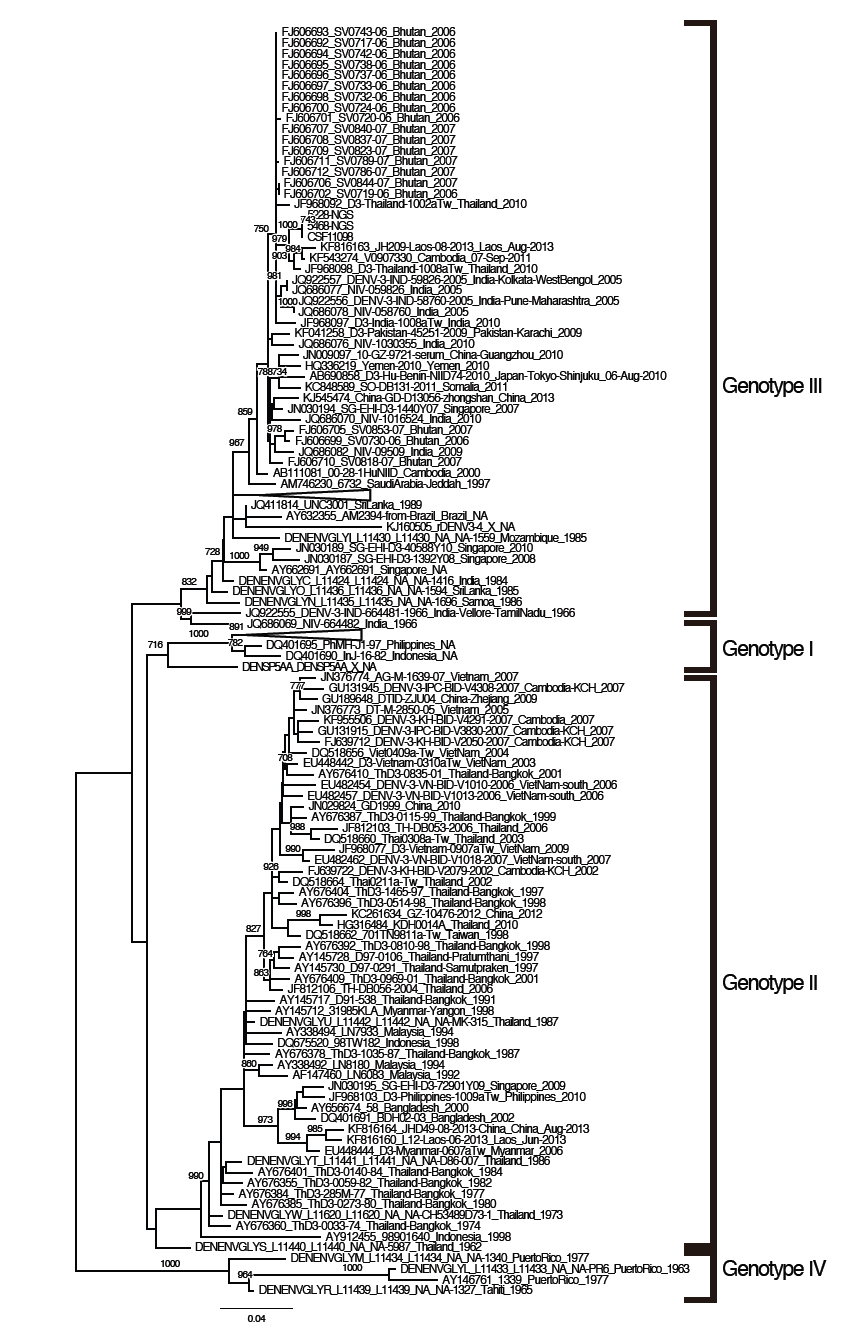

Genotype III

**Supplementary Figure 1.** ML-phylogenetic tree based on the envelope gene sequence of DENV3 isolates. The envelope sequence of DENV3 (CSF11098) isolated from the CSF of our case-patient in Hai Phong, Vietnam in 2013 was compared with those of DENV3 (5228 NGS and 5468 NGS) isolated from serum samples of other patients in the same place and in the same year. The strain names of these three DENV3 isolates were enclosed in red square. This fragment of CSF derived DENV3 was also compared with others homologous sequences of DENV3 in GenBank database from different geographical regions. Bootstrap values over 800 > of 1000 repeats are shown at the nodes. Labels of strains conform to the following format: (GenBank accession nos)_(Strain name)_(Country-region)_(Year of isolation).


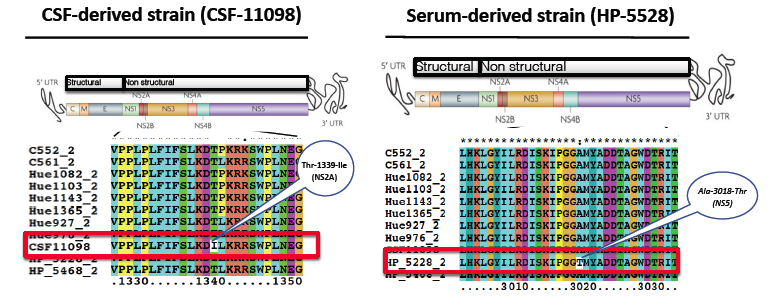


**Supplementary Figure 2.** *Thr-1339-Ile* mutations in non-structural regions 2A (NS2A) sequences in CSF-derived DENV3 strain (CSF- 11098) and, Ala-3018-Thr mutation in non-structural regions 5 (NS5) sequences in serum-derived DENV3 strain (HP-5528).

**Supplementary Table 1.** Characteristics of the primers and probes used in one-step real time PCR.

| **Name** | **Sequence (5’-3’)** | **Position^1^** | **Nucleotide** |
| --- | --- | --- | --- |
| DENV3-probe | 5'-FAM-AgATTTTgTggAAggYCT MGB(NFQ)-3’ | 928 | 18 |
| DENV3-Forward | 5’-CYTGGWTGTCDRCYGARGGAG-3’ | 726 | 21 |
| DENV3-Reverve | 5’-TGCACCACTTTTCCCTCTAT-3’ | 1271 | 20 |

FAM, carboxyl fluorescein; MGB, minor groove binding; NFQ, non-fluorescein quencher. ^1^Genome positions are given according to DENV3-NCBI reference sequence (Genbank acc. no. EU529696).

**Supplementary Table 2.** Characteristics of the primers used to generate standard curves for one-step real-time PCR.

| **Name** | **Sequence (5’-3’)** | **Position^1^** | **Nucleotide** |
| --- | --- | --- | --- |
| D3-F-902 | 5’-CCATGACAATGAGATGTGTGGGAGTGGGAAA-3’ | 891 | 31 |
| F2-768-D3-2014 | 5’-CYTGGWTGTCDRCYGARGGAG-3’ | 726 | 21 |
| D3-1307-2R | 5’-TGCACCACTTTTCCCTCTAT-3’ | 1271 | 20 |
| D3-1684-3R | 5’-GCTCCCTCTTGCGATCCAAGG-3’ | 1648 | 21 |
